# Averaging Pixel Current Adjustment Technique for Reducing Fixed Pattern Noise in the Bolometer-Type Uncooled Infrared Image Sensor

**DOI:** 10.3390/s19071653

**Published:** 2019-04-06

**Authors:** Sang-Hwan Kim, Byoung-Soo Choi, Jimin Lee, Junwoo Lee, Jewon Lee, Jae-Hyoun Park, Kyoung-Il Lee, Jang-Kyoo Shin

**Affiliations:** 1 School of Electronics Engineering, Kyungpook National University, Daegu 41566, Korea; shkim7@knu.ac.kr (S.-H.K.); bschoi@ee.knu.ac.kr (B.-S.C.); jmLee@ee.knu.ac.kr (J.L.); junwoolee@knu.ac.kr (J.L.); jewonlee@knu.ac.kr (J.L.); 2 Korea Electronics Technology Institute, Seongnam-si 13509, Korea; jhpark@keti.re.kr (J.-H.P.); leeki@keti.re.kr (K.-I.L.)

**Keywords:** uncooled infrared image sensor, fixed pattern noise, current calibration

## Abstract

In this paper, we propose an averaging pixel current adjustment technique for reducing fixed pattern noise (FPN) in the bolometer-type uncooled infrared image sensor. The averaging pixel current adjustment technique is composed of active pixel, reference pixel, and calibration circuit. Polysilicon resistors were used in each active pixel and reference pixel. Resistance deviation among active pixels integrated with the same resistance value cause FPN. The principle of the averaging pixel current adjustment technique for removing FPN is based on the subtraction of dark current of the active pixel from the dark current of the reference pixel. The subtracted current is converted into the voltage, which contains pixel calibration information. The calibration circuit is used to adjust the calibration current. After calibration, the nano-ampere current is output with small deviation. The proposed averaging pixel current adjustment technique is implemented by a chip composed of a pixel array, a calibration circuit, average current generators, and readout circuits. The chip was fabricated using a standard 0.35 μm CMOS process and its performance was evaluated.

## 1. Introduction

The bolometer-type uncooled infrared image sensor has recently been researched in various fields such as defense, medical care, and non-destructive testing [1,2,3]. Uncooled infrared image sensors have many advantages in terms of low power consumption, easy integration, and low cost [4,5,6,7,8,9,10,11]. Among the various uncooled infrared image sensors, the bolometer sensor based on micromachining technology has a higher temperature coefficient of resistance than other sensors. The response of a bolometer sensor, which is one type of thermal detector, is slow but it does not depend on the wavelength of infrared light and it can be used at room temperature. Therefore, there is no need for a thermoelectric cooler that consumes additional power and volume. In addition, various readout integrated circuits (ROICs) for bolometer-type uncooled infrared image sensors using a standard CMOS process have been developed [12,13,14]. Among the various ROICs, the capacitive transimpedance amplifier (CTIA) is widely used to detect the signal output current of pixels [15]. The bolometer sensor based on micromachining technology is compatible with the standard CMOS process, but low linearity and high noise are disadvantages.

Resistance deviation among the resistors integrated in the active pixel and reference pixel causes serious fixed pattern noise (FPN). In a readout circuit and a signal processing circuit integrated using a standard CMOS process, a method for compensating for these deviations is necessary in order to obtain an efficient infrared image. Various methods have been researched to solve the problem of the deviation between resistors integrated in the active pixel and reference pixel [16,17,18].

In this research, in order to compensate the resistance deviation occurring among the pixels of the bolometer-type uncooled infrared image sensor, the averaging pixel current adjustment technique was used, which reduces the dark current by averaging the currents generated in the bolometers of reference pixels in column readout circuits. For verifying the proposed averaging pixel current adjustment technique, the output nodes of each reference pixel are all tied. As a result, the output current of the reference pixel in which the light receiving portion is blocked to avoid being affected by the incident light is averaged. The bolometer resistor in the active pixels and reference pixels has a dark current component. When the current from each active pixel array is subtracted from the current from the reference pixel, the dark current of the active pixel can be removed. The averaging pixel current adjustment technique consists of a reference pixel, active pixel, calibration circuit, and readout circuit. The entire chip based on the averaging pixel current adjustment technique was fabricated using a 0.35 μm standard CMOS process and its characteristics were analyzed.

## 2. Principle of Pixel Averaging Pixel Current Adjustment Technique

**Figure 1:**
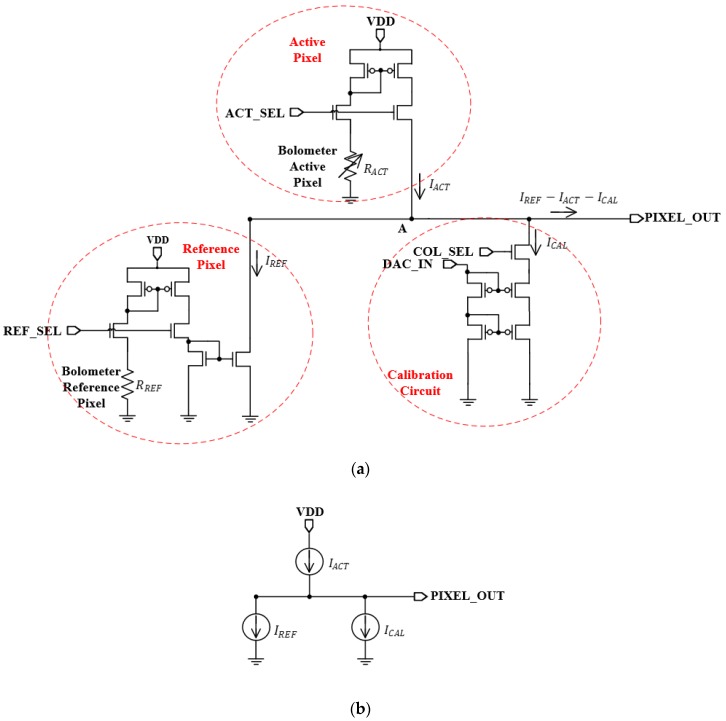
(**a**) Schematic of averaging pixel current adjustment technique (**b**) modeling of averaging pixel current adjustment technique.

Figure 1a shows the schematic of the averaging pixel current adjustment technique. Each unit pixel is composed of an active pixel, reference pixel and calibration circuit. The bolometer resistors in the active pixel and reference pixel were integrated using resistors. Figure 1b shows the modeling of the averaging pixel current adjustment technique. Kirchhoff’s Current Law (KCL) is applied to Node A in Figure 1a, and the output dark current of the active pixel ($I_{ACT}$) can be subtracted from the output dark current of the reference pixel ($I_{REF}$). Additionally, the output current of the calibration circuit ($I_{CAL}$) can be adjusted to account for reducing the dark current component. The current obtained by subtracting the dark current of the active pixel from the dark current of the reference pixel ($I_{REF}-I_{ACT}$) has deviation information that is generated by the resistance of the active pixel. The principle of detection and calibration for dark current is explained as follows:

(1) $$I_{REF}\propto \frac{1}{R_{REF}}$$

(2) $$I_{ACT}\propto \frac{1}{R_{ACT}}$$

(3) $$I_{ACT}+\Delta {I^{\prime }}_{ACT}\propto \frac{1}{R_{ACT}+\Delta {R^{\prime }}_{ACT}}$$

(4) $$I_{REF}-I_{ACT}+\Delta {I^{\prime }}_{ACT}-I_{CAL}≜\mathrm{nA}\;\mathrm{order}$$

where $\Delta {R^{\prime }}_{ACT}$ is the active bolometer resistance that is occurs by a process deviation and $\Delta {I^{\prime }}_{ACT}$ is the deviation of the output dark current of the active pixel. In a unit pixel, the output dark current of the reference pixel ($I_{REF}$) is inversely proportional to the reference bolometer resistance ($R_{REF}$) and the output dark current of the active pixel ($I_{ACT}$) is inversely proportional to the active bolometer resistance ($R_{ACT}$). The $I_{ACT}$ changes when the $R_{ACT}$ in all the unit pixels is changed by the process deviation. Therefore, the $\Delta {I^{\prime }}_{ACT}$ that causes the FPN is generated. Additionally, by reducing the $\Delta {I^{\prime }}_{ACT}$ using the current of the calibration circuit ($I_{CAL}$), the final output current can be reduced to nA order. 

The deviation information is converted to voltage and digital codes through a current–voltage converter and an analog–digital converter (ADC), respectively. This deviation information causes the FPN. The $I_{CAL}$ proportional to the output code of the analog digital converter reduces the $I_{ACT}$. At this time, if $I_{ACT}$ is designed to always be larger or smaller than $I_{REF}$, the direction of $I_{CAL}$ can be determined in one direction. When the output code of the ADC is controlled so that $I_{CAL}$ is almost equal to $I_{REF}-I_{ACT}$, the pixel current offset due to the deviation of each active bolometer resistance is eliminated. Considering the characteristics of the current voltage converter, $I_{CAL}$ is controlled so that the output current offset of each active pixel is nA order.

**Figure 2:**
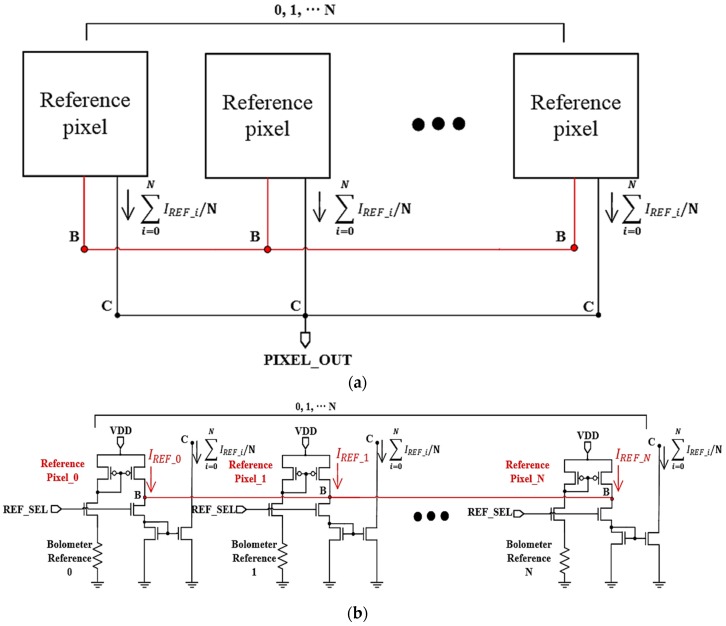
(**a**) Block diagram of averaging current generator using reference pixel, (**b**) schematic diagram of averaging current generator using reference pixel.

In order to obtain the deviation information based on the resistance of the active pixel using the proposed averaging pixel current adjustment technique, the resistance of the reference pixel must be fixed without any deviation. However, not only the resistor of the active pixel has a process deviation, but also the resistor of the reference pixel has a process deviation. Figure 2a,b show a block diagram and a schematic diagram of the averaging current generator using the reference pixel, respectively. The average current generator was used to remove the offset current caused by resistance deviation among the resistors integrated in the active pixel. The principle for removing the offset current of the active pixel using the average current generator is as follows. First, output dark currents ($I_{REF\_0}$, $I_{REF\_1}$, $I_{REF\_N}$) are generated at Node B of the reference pixels in each unit pixel. At this time, the output dark currents do not have uniform values due to the process deviation of the reference bolometer in each reference pixel. Second, output Node B of all the reference pixels is shared so that the output dark currents have an averaged value. Therefore, the average current ($\sum_{i=0}^{N}I_{REF\_i}/N$) at node C can be obtained. Finally, the average current is used as the output dark current of the reference pixel ($I_{REF}$) in Figure 1a. When the number of reference pixels increases, the average current converges to the average value. In this way, when the active pixel current ($I_{ACT}$) is subtracted using the average current, FPN is removed.

**Figure 3:**
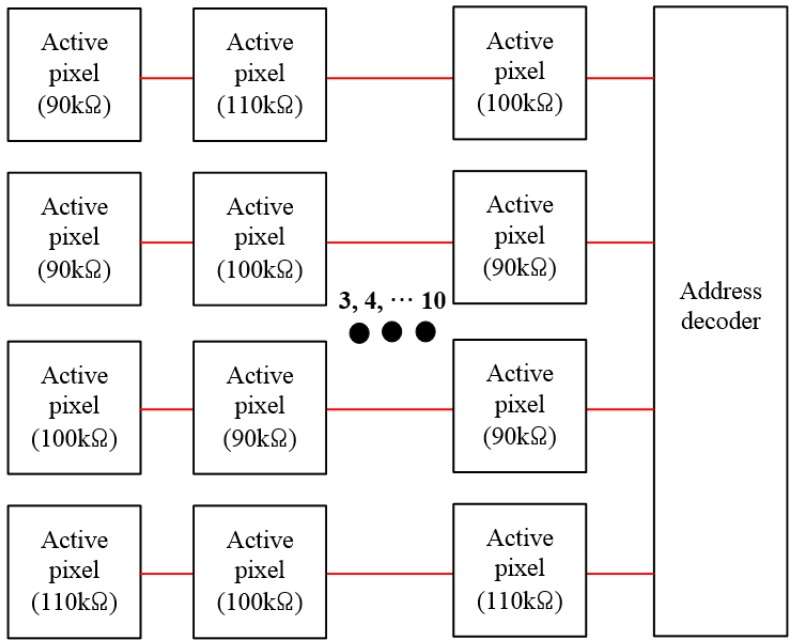
Resistance distribution of the active pixel.

Figure 3 describes the resistance distribution of the active pixel. A bolometer with resistance deviations included for each active pixel was implemented with 90 kΩ, 100 kΩ and 110 kΩ resistors based on 100 kΩ resistances, taking into account 10% resistance deviation in the process. The array is composed of 4 × 11 active pixels. The bolometer included in the reference pixel consists of only 110 kΩ resistors, and it was fabricated using the standard CMOS process.

**Figure 4:**
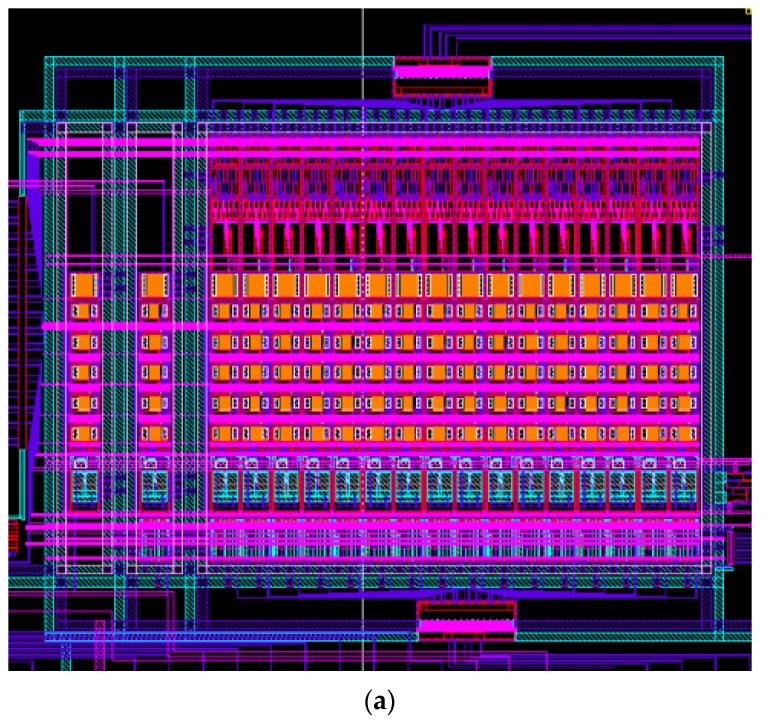

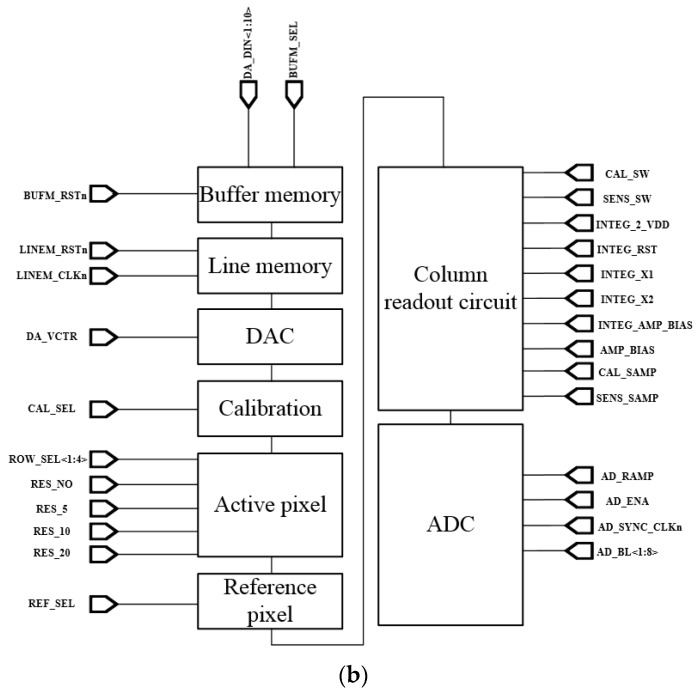
(**a**) Layout of designed readout circuit (**b**) each column block diagram of designed chip. DAC: digital analog converter; ADC: analog digital converter.

Figure 4a shows the layout of the designed readout circuit and Figure 4b shows each column block diagram of the designed chip. The entire chip is based on column parallel structure. Each column of the entire chip is composed of buffer memory, line memory, a digital–analog converter (DAC), a calibration circuit, an active pixel, a reference pixel, a column readout circuit, and an ADC. The buffer memory, line memory, and DAC are used to control the calibration current ($I_{CAL}$), which is a nano-ampere current. The column readout circuit converts current generated in a unit pixel into a voltage using an integrator based on a two-stage amp. 

**Figure 5:**
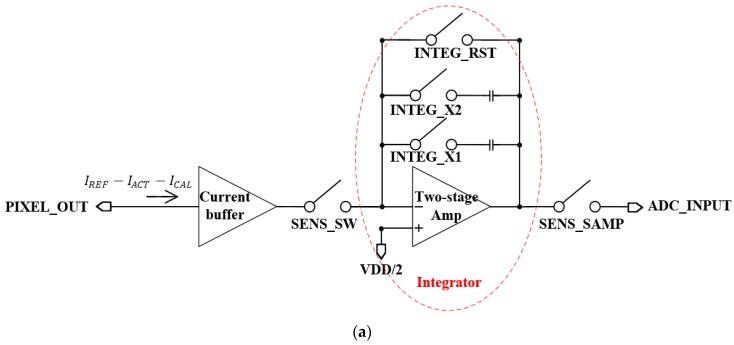

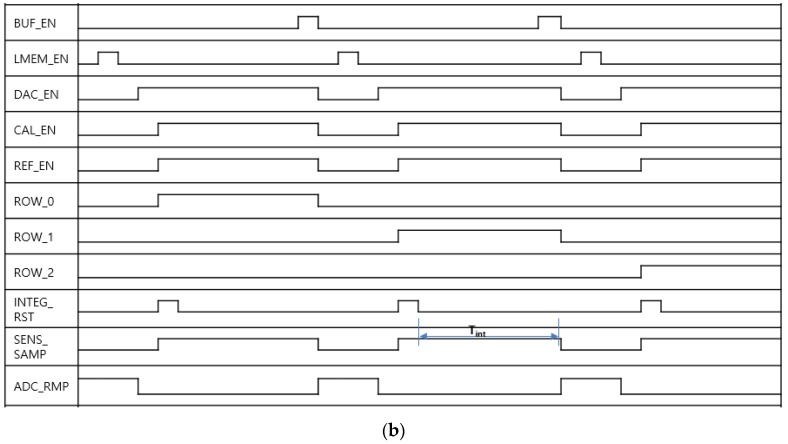
(**a**) Schematic of column readout circuit with integrator for current to voltage conversion, (**b**) timing diagram of entire chip.

Figure 5a shows the schematic of the column readout circuit with integrator for current-to-voltage conversion, and Figure 5b shows the timing diagram of the entire chip. The column readout circuit is composed of a current buffer for the current generated in the unit pixel, an integrator, a switch to control the gain of the integrator, a switch to reset the integrator, and a switch for sampling and selecting. The integrator is designed to control the output voltage gain based on a two-stage amplifier. The entire chip is based on a column parallel structure. Therefore, after the reset pulse (INTEG_RST) of the integrator is applied, the integrator output voltage is sequentially obtained for each row of the pixel. The integration time ($T_{int}$) is the time from the reset of the integrator to the end of the operation of the sampling switch.

## 3. Measurement Results and Discussion 

In the case of the signal processing circuit of the bolometer-type uncooled infrared image sensor, it is important that the signal electrons generated by the infrared radiation or dark current of each pixel obtain the same output voltage for a certain integration time by the integrator. In order to verify the averaging pixel current adjustment technique, we compared the output current of the un-calibrated pixel and the integrator output voltage generated by the output current of the calibrated pixel according to the integration time.

**Figure 6:**
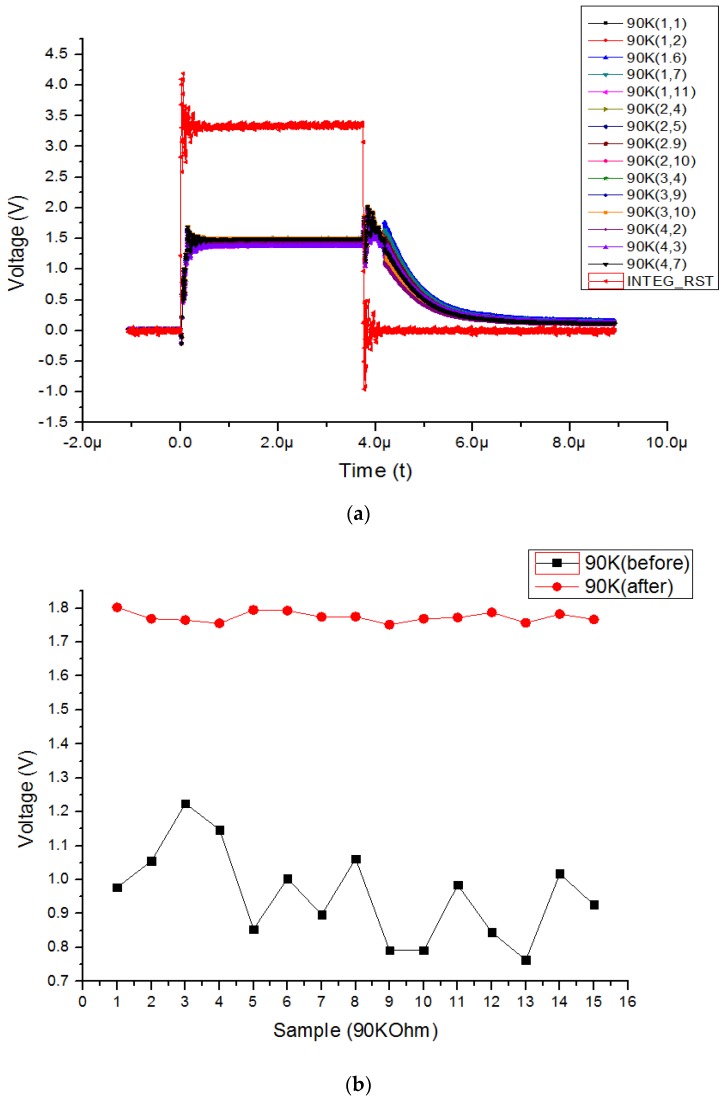
(**a**) The 90 kΩ active pixel output voltage according to integrator reset voltage before calibration (**b**) experimental results before and after applying proposed adjustment technique to the 90 kΩ active pixel.

Figure 6a shows the 90 kΩ active pixel output voltage according to the integrator reset voltage before calibration, and Figure 6b shows the experimental results before and after applying the proposed averaging pixel current adjustment technique to the 90 kΩ active pixel. The entire pixel array is composed of 4 × 11 pixels. The active pixel integrated in the entire pixel array has a resistance of 90 kΩ and 110 kΩ, which has a 10% process deviation, randomly integrated based on 100 kΩ. Only a 110 kΩ resistor is integrated in the reference pixel. A 90 kΩ resistor has a 10% process deviation even at the same resistance value. After the reset of the integrator, the output voltage decreases to VDD/2 (1.65V) according to the input current of the integrator generated by the resistor integrated in the active pixel. It was confirmed that the output voltage of the integrator before calibration was different for each active pixel due to the process deviation of the resistance. However, it was also confirmed that the output voltage of the integrator was nonlinear at the initial point after the reset of the integrator. Due to clock feedthrough by the integrator reset switch, the nonlinear output voltage is generated. Furthermore, the integration time is decreased as the deviation of the resistance of the active pixel and resistance of the reference pixel becomes larger. Therefore, it is assumed that the active pixel to which the adjustment technique is applied increases the integration time compared with the case where it is not. When the adjustment technique is applied in Figure 6b, it is confirmed that the uniform output voltage can be obtained even if the process deviation occurs at each 90 kΩ resistor in the active pixel.

**Figure 7:**
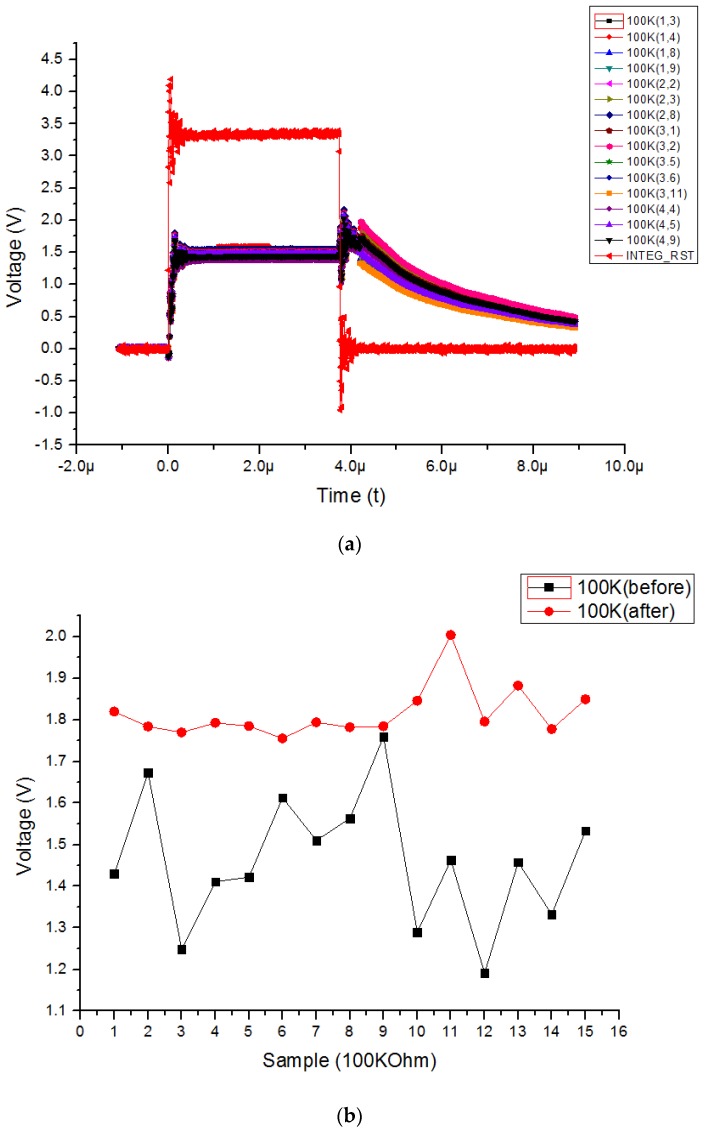
(**a**) The 100 kΩ active pixel output voltage according to integrator reset voltage before calibration (**b**) experimental results before and after applying proposed adjustment technique to the 100 kΩ active pixel.

Figure 7a shows the 100 kΩ active pixel output voltage according to the integrator reset voltage before calibration and Figure 7b shows the experimental results before and after applying the proposed averaging pixel current adjustment technique of the 100 kΩ active pixel. Compared with the output voltage of the integrator when the resistance of the active pixel 90 kΩ, the integration time is relatively increased. In addition a comparison with the output voltage of the integrator when the active pixel is 90 kΩ, confirms that the error remains after calibration. Compared with the case in which the resistance of the active pixel is 90 kΩ and the resistance of the active pixel is 100 kΩ, the resistance of the reference pixel is 110 kΩ, so the $I_{REF}-I_{ACT}$ current is relatively low. Therefore, not only the amount of current to be calibrated but also the absolute amount of deviation is reduced. A comparison of before and after the calibration, confirms that the red line increases to about 2 V in a Sample 11 of Figure 7b because an instantaneous clock feedthrough occurs when the reset switch of the integrator is opened. The reason the difference between the voltage before and after the calibration is small in the case of Sample 9 of Figure 7b is that the deviation of the active pixel resistance is about +10%, which is similar to the resistance of the reference pixel. However, the deviation of the integrator output voltage due to the resistance deviation based on 100 kΩ is shown in Figure 7a. In addition, it is confirmed that the resistance deviation among active pixels is also reduced by applying the averaging pixel current adjustment technique.

**Figure 8:**
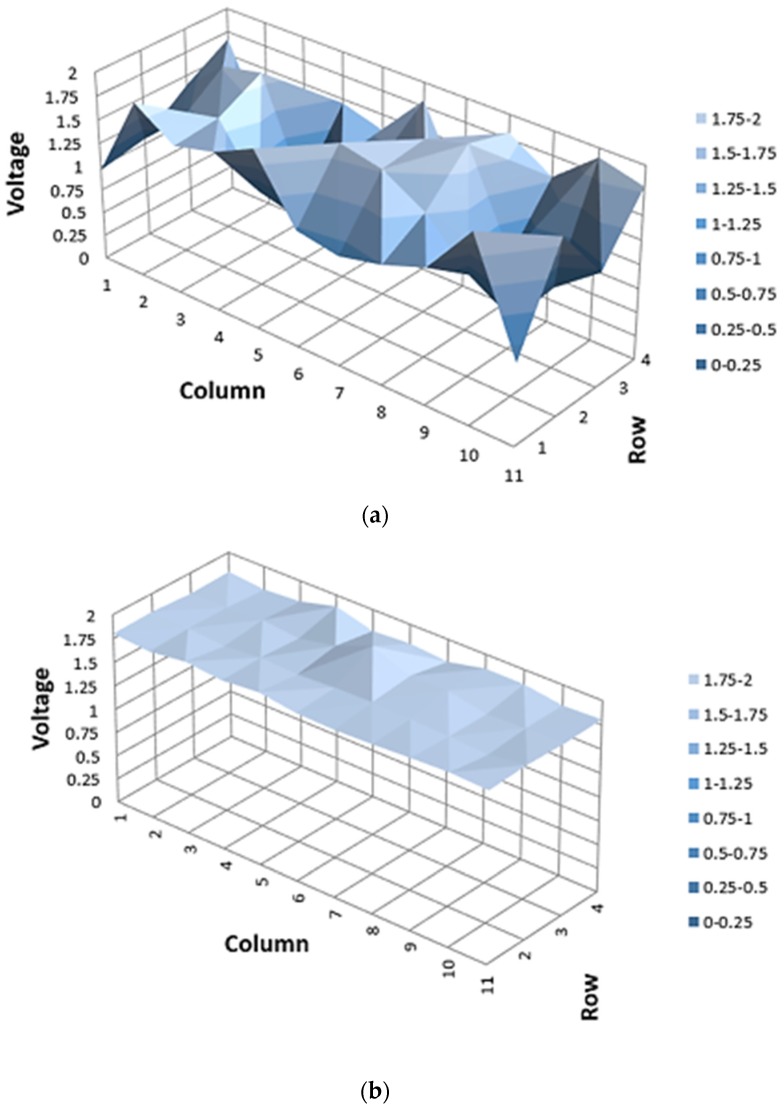
3D graph of before (**a**) and after (**b**) applying proposed adjustment technique to the entire array.

A 3D graph of before and after the proposed averaging pixel current adjustment technique of the entire array was applied is presented in Figure 8. In the 4x11 array where the resistance of the active pixel is integrated 90 kΩ, 100 kΩ, and 110 kΩ randomly, the voltage which is on the *Z*-axis, is the output voltage of the integrator. Figure 8a shows that before applying the averaging pixel current adjustment technique, the standard deviation of the integrator output voltage due to the output current among the active pixels is 0.385. The standard deviation is the deviation amount of each active pixel when the output voltage of the active pixel is averaged. In addition, the standard deviation is FPN occurring between the active pixels due to the resistor’s process deviation. Figure 8b is a 3D graph showing after the averaging pixel current adjustment technique is applied. Compared with Figure 8a, the slope is relatively gradual and confirmed by measurement. The standard deviation between each active pixel after applying the averaging pixel current adjustment technique is 0.043. Therefore, it is confirmed that the proposed averaging pixel current adjustment technique reduces the FPN by about 34%.

## 4. Conclusions

In this paper, we proposed an averaging pixel current adjustment technique for reducing FPN in the bolometer-type uncooled infrared image sensor. In order to reduce the resistance deviation causing the FPN among pixels of the bolometer-type uncooled infrared image sensor, an averaging pixel current adjustment technique was successfully implemented using a standard 0.35 μm CMOS process. According to the experimental results, it was confirmed that the standard deviation of the output voltage according to the resistance of the active pixel was reduced by about 34% when the active pixel output voltages before and after the calibration were compared. Therefore, it was concluded that the output offset current according to the deviation of the bolometer resistance of each pixel was reduced by controlling the output code of the ADC using the proposed technique. We expect the proposed averaging pixel current adjustment technique to be effectively applied to the bolometer-type uncooled infrared image sensor for reducing FPN.
